# Selective activation of antioxidant resources and energy deficiency in Marinesco–Sjögren syndrome fibroblasts as an adaptive biological response to Sil1 loss

**DOI:** 10.1038/s41598-025-96467-9

**Published:** 2025-04-11

**Authors:** Valeria Panella, Francesca Potenza, Carla Tatone, Lorenza Speranza, Fernanda Amicarelli, Michele Sallese

**Affiliations:** 1 Department of Medicine and Aging Sciences, “G. d’ Annunzio” University of Chieti-Pescara, Chieti, 66100 Italy; 2 Department of Innovative Technologies in Medicine and Dentistry, “G. d’ Annunzio” University of Chieti-Pescara, Chieti, 66100 Italy; 3 Center for Advanced Studies and Technology (CAST), “G. d’ Annunzio” University of Chieti-Pescara, Chieti, 66100 Italy; 4 Department of Life, Health and Environmental Sciences, University of L’Aquila, L’Aquila, 67100 Italy

**Keywords:** Neurodegenerative disease, Ataxia, Mitochondria, Energy deficiency, Catalase, Superoxide dismutase, ROS, Sil1, Biochemistry, Cell biology

## Abstract

Marinesco–Sjögren syndrome (MSS) is a neuromuscular disease which presents with ataxia, muscle weakness and cataracts. This syndrome is typically caused by mutations in *SIL1* gene, an ER co-chaperone that disrupts protein folding. Although it is known that accumulation of misfolded proteins in the ER profoundly affect reduction–oxidation (redox) homeostasis and energy production, the possible role of these processes in MSS was not investigated to date. In patient-derived fibroblasts, both maximal mitochondrial respiration and mitochondrial ATP production rates were diminished, while the glycolytic fraction remained unaffected. Catalase and superoxide dismutase activities were increased, while glutathione peroxidase and glutathione reductase were decreased. Oxidative damage to lipids, proteins, and DNA was comparable or even lower to that observed in control cells. Similar alterations were observed in the muscle tissue of the woozy mouse model of MSS. In conclusion, we identified a mitochondrial energy deficit and an adaptive cellular mechanism that effectively manage oxidative stress in Sil1-deficient cells.

**Supplementary Information:**

The online version contains supplementary material available at 10.1038/s41598-025-96467-9.

## Introduction

Misfolding diseases are a class of disorders resulting from the accumulation of aberrantly folded proteins. These misfolded proteins can form aggregates that impair cellular function and lead to tissue damage, particularly in neurodegenerative disorders. The accumulation of these misfolded proteins also triggers a cellular stress response known as unfolded protein response (UPR). The UPR aims to restore endoplasmic reticulum (ER) homeostasis through three transmembrane proteins, protein kinase RNA-Like ER Kinase (PERK), inositol-requiring enzyme 1 (IRE1) and activating transcription factor 6 (ATF6), that reduce the rate of protein synthesis, increase the ER folding potential and protein degradation capacity (ER-associated degradation, ERAD). Protein folding involves the formation of intermediate and terminal disulfide bridges assisted by the oxidoreductase-isomerase called protein-disulfide isomerase (PDI) or its homologs ER–resident protein 57 (ERP57) and ERP72^1^. After catalysing disulfide bridge formation on newly synthesized proteins, the oxidoreductases are in a reduced form and must be re-oxidized to continue their work^1,2^. Several proteins participate in oxidoreductase reoxidation, including ER oxidoreductase 1 alpha (ERO1α), which produces hydrogen peroxide as a byproduct. It was estimated that about 25% of cellular reactive oxygen species (ROS) are generated by the ER, while the rest is produced by mitochondria. In particular, a small amount of electrons flowing through mitochondrial complexes I to III physiologically escapes and generates superoxide anions. However, the amount of ROS generated by the mitochondria can increase when the electron flow is impaired^3–5^. Interestingly, under conditions of ER stress and UPR, the ATP requirement in the ER is higher and is mainly supplied by the mitochondria rather than the glycolytic pathway^6–8^.

The generation of intracellular ROS is a physiological event necessary for multiple cellular functions, including cell signalling and defence against pathogens, provided there is a balance between ROS produced, ROS utilised and ROS scavenged by antioxidant systems^9^. Cells have several protective systems to prevent damage caused by ROS, these include small molecules (e.g. ascorbic acid, glutathione, tocopherols) and enzymes such as catalase (CAT), superoxide dismutase (SOD), glutathione peroxidase (GPx) and glutathione reductase (GR)^9^. Imbalance of intracellular ROS causes oxidative stress that damages membrane lipids, proteins, and DNA and can lead to UPR activation^10^.

On the other hand, when ER folding is challenged, ROS production increases due to reiterated folding attempts^11^. Notably, both activation of the UPR and altered ROS production were reported in misfolded diseases like tauopathies including Alzheimer’s disease^12–15^, α-synucleinopathies including Parkinson’s disease^16^, Huntington’s disease^17,18^, and prion disease^19,20^. Marinesco–Sjögren’s syndrome (MSS) is a rare genetic disorder characterised by cerebellar ataxia caused by degeneration of Purkinje neurons, muscle weakness, hypotonia and ocular cataracts, although several other symptoms have been reported, including bone abnormalities and cognitive impairment. So far, Sil1 has been recognized as the gene responsible for about half of the MSS cases, while the trigger for the remaining ones is still unknown. Sil1 is a nucleotide exchange factor that assists the functioning of the ER chaperone known as binding immunoglobulin protein (Bip). Loss of Sil1 impairs protein folding activity by BiP, resulting in altered ER homeostasis and activation of the UPR. The activation of the ER stress response in MSS has been described in detail and most likely participates in the pathological loss of Purkinje neurons and muscle cells^21–23^. Whereas, the possible contribution of ROS in MSS pathology was not investigated. However, Sil1 has been shown to have antioxidant activity against BiP in Saccharomyces cerevisiae^24^.

In the present study, by analysing energy metabolism, antioxidant mechanisms and ROS in patient-derived fibroblasts, we found that the production of glycolytic ATP is similar to that of control fibroblasts, while ATP generated by mitochondria is significantly reduced. Concerning antioxidant enzymes, CAT and SOD activities were increased, while those of GPx and GR were reduced in these patient-derived fibroblasts. This new set-up of antioxidant systems seems adequate to keep ROS levels low and protect patient cells from oxidative damage. In fact, several biomarkers of oxidative damage, including malondialdehyde (MDA), 4-hydroxynonenal (4-HNE), protein carbonyl content (PCC) and phosphorylation of the histone H2AX (γ-H2AX) remained unchanged or even reduced. These results were confirmed in the Woozy mouse quadriceps, a widely used representative mouse model of human MSS.

## Results

### Metabolic changes in fibroblasts isolated from a patient affected by MSS

**Fig. 1:**
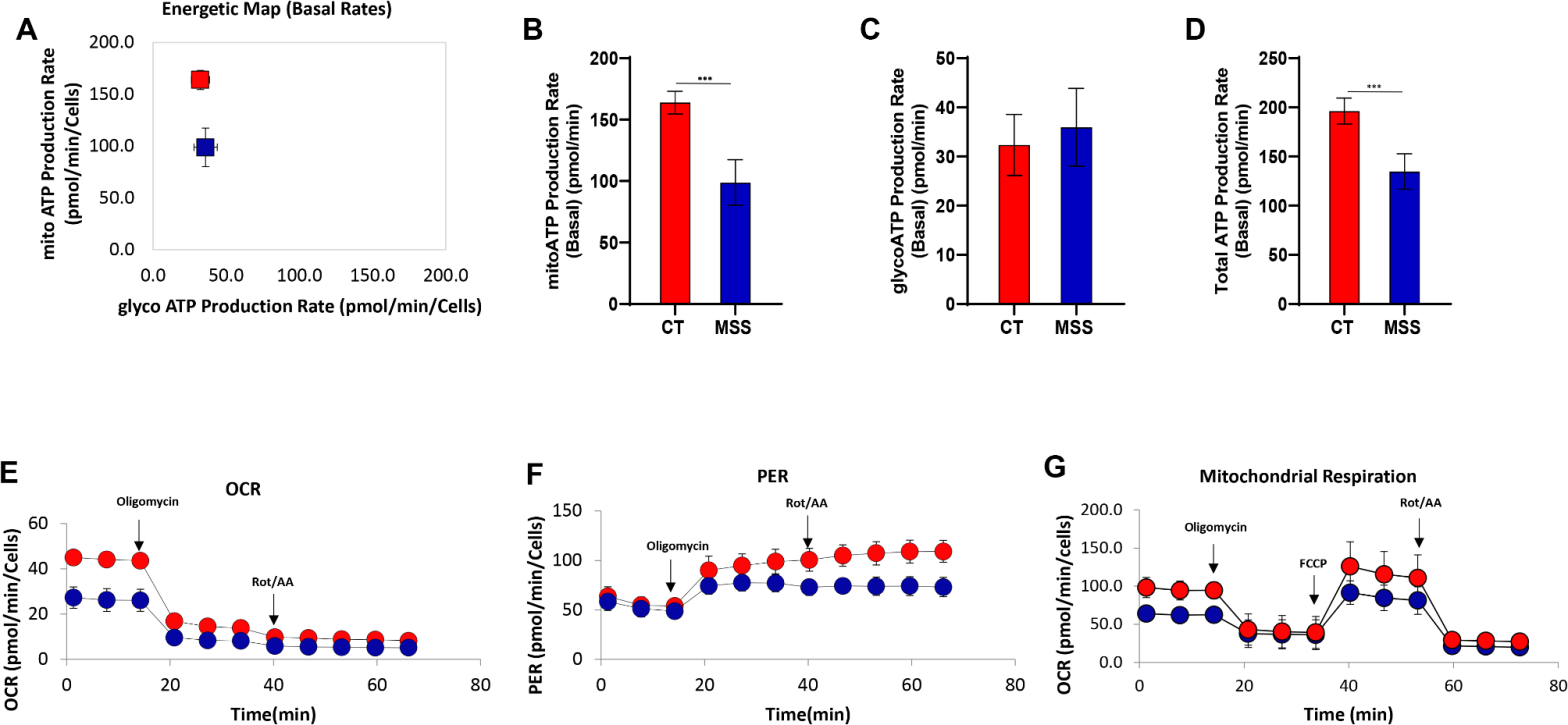
Energetic profile of MSS fibroblasts measured with Seahorse using ATP rate assay. (**A**–**C**) Control fibroblasts (CT) and patient-derived fibroblasts (MSS) energetic map and ATP production from mitochondrial and glycolytic pathways. The histograms show comparable levels of ATP from the glycolytic pathway and a decrease in ATP from oxidative phosphorylation in MSS cells. (**D**) Quantification of total ATP production shows a decrease in MSS cells. (**E**,**F**) OCR and PER graphs show both decreased levels in MSS cell. (**G**) OCR measurements in cells treated with FCCP showed reduced maximal mitochondrial respiration in patient-derived fibroblasts. Statistical analysis was conducted using an unpaired *t*-test with Welch’s correction (***P < 0.001).

The loss of Sil1 in MSS leads to the accumulation of unfolded proteins in the ER and the activation of unfolded protein response^22,25,26^. Such a situation requires more ATP, which is largely produced by oxidative phosphorylation in mitochondria and transferred to the ER through mitochondria-associated membranes (MAMs)^6,7^. Through proteomic analysis, we previously reported an upregulation of proteins involved in both beta-oxidation and the tricarboxylic acid cycle in patient-derived fibroblasts, with the possible purpose of increasing oxidative phosphorylation and, consequently, mitochondrial ATP production^26^. To assess whether mitochondria in patient fibroblasts actually generated more ATP than control fibroblasts, we used Agilent’s Seahorse. Patient-derived fibroblasts had a mitochondrial ATP production rate much lower than control fibroblasts while ATP generated by the glycolysis was comparable between the two primary cell lines (Fig. 1A–C). As a result, the overall ATP production was significantly reduced (Fig. 1D). These results were obtained by measuring the proton efflux rate (PER), which is mainly due to glycolytic activity, and the oxygen consumption rate (OCR), which is an indicator of mitochondrial activity (Fig. 1E, F). Measurements were performed both at steady state and after oligomycin and rotenone/antimycin treatment to assess how much of the mitochondrial respiration is engaged in ATP synthesis and to assess non-mitochondrial respiration (Fig. 1E, F). Finally, treatment with carbonyl cyanide 4-(trifluoromethoxy)phenylhydrazone (FCCP) revealed that maximal mitochondrial respiration was lower in patient-derived fibroblasts compared to controls (Fig. 1G). The decrease in maximal respiration and mitochondrial ATP production align with the aberrant mitochondria reported in skeletal muscle of MSS patients and woozy mice^27,28^.

### The antioxidant enzymes CAT and SOD are upregulated in MSS fibroblasts

Both the impairment of mitochondrial functions (see above) and activation of the ER stress response can lead to the formation of excessive amounts of ROS^29^. Furthermore, our previous omics analysis of fibroblasts isolated from a MSS patient identified an upregulation of CAT and both the isoforms of SOD (Cu-ZnSOD, MnSOD)^26,30^. Based on these indications, we decided to investigate the presence and possible consequences of oxidative stress in these fibroblasts.

**Fig. 2:**
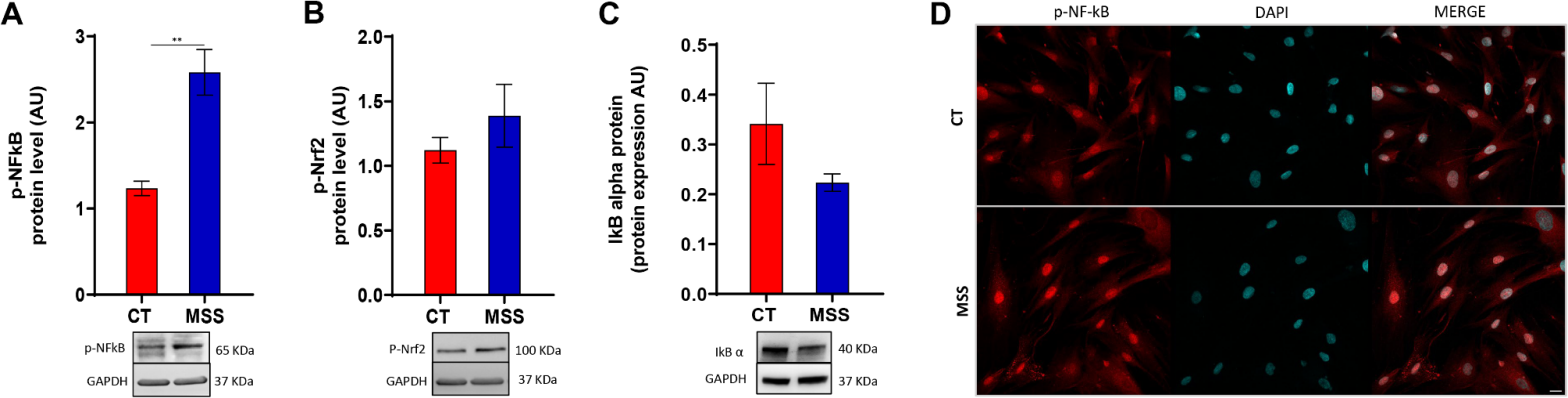
Study of Nrf2 and NFkB activation in MSS fibroblasts. (**A**,**B**) Western blot showing the levels of Nrf2 (**A**) and NF-kB p65 (**B**) phosphorylation in patient-derived fibroblasts (MSS) and controls (CT). In both cases the phosphorylated form represents the transcriptionally active factor. (**C**) Western blot analysis of IκBα revealed a decreased protein level in MSS compared to CT cells. Statistical analysis was performed using an unpaired *t*-test with Welch’s correction (**P < 0.01). (**D**) IF analysis of the intracellular distribution of phosphorylated NF-kB p65. CT and MSS cells were plated to 70% confluency, and the following day, cells were fixed and processed for IF. NF-κB p65 staining is shown in red, and DAPI in blue. Merged images are also provided. Scale bar: 20 μm.

**Fig. 3:**
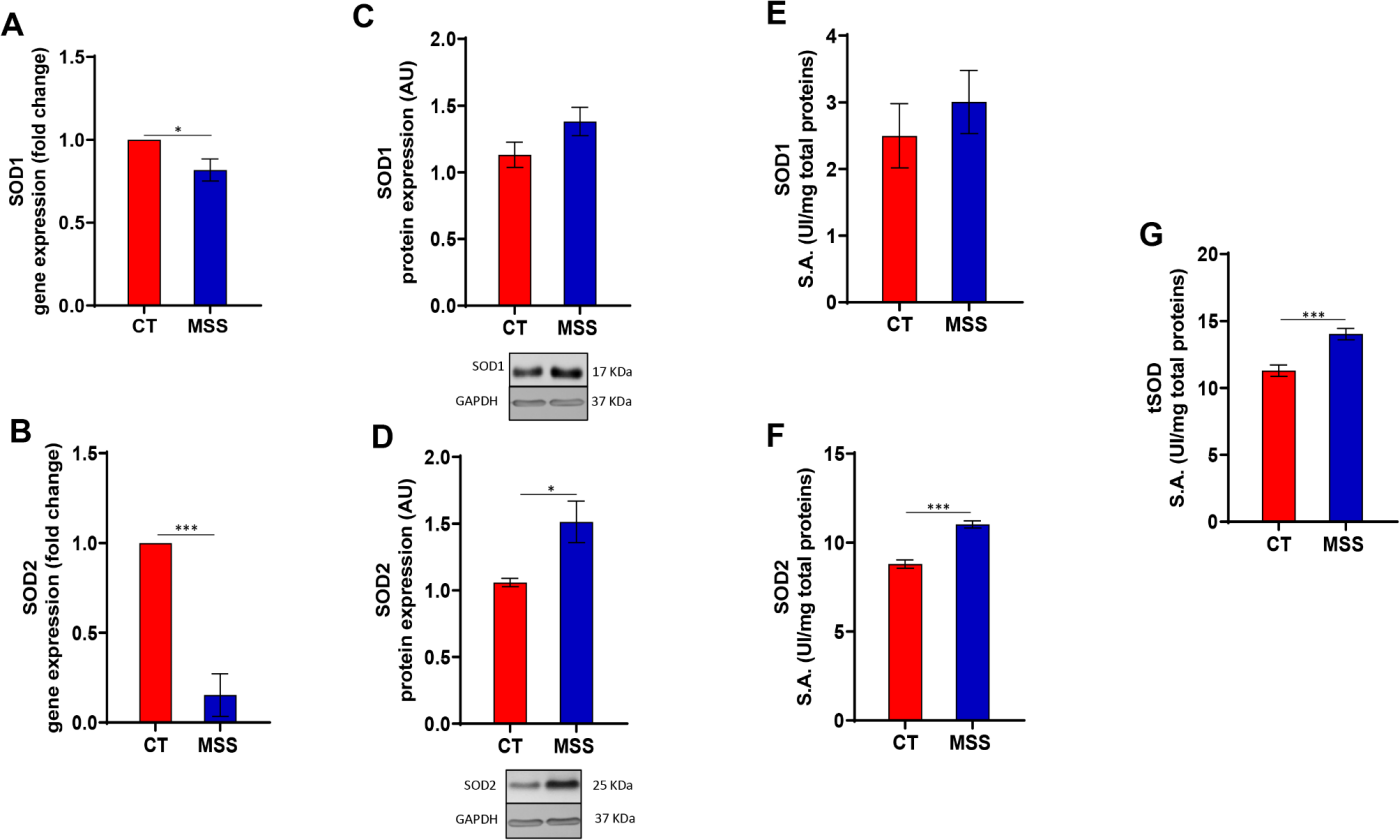
Activation of the antioxidant response in MSS fibroblasts. (**A**,**B**) Both graphs show gene expression of SOD1 and SOD2 measured by real-time PCR using ΔΔCt method. (**C**,**D**) Western blot analysis of SOD1 and SOD2 in patient-derived fibroblasts (MSS) and controls (CT) shows a significant increase of SOD2 in MSS cells compared to CT cells. (**E**,**F**) The enzymatic activity of both antioxidant enzymes (SOD1 and SOD2) shows a significant raise in the pathological condition as well as the total SOD activity (G). Statistical analysis was performed using an unpaired *t*-test with Welch’s correction (*P < 0.05, ***P < 0.001).

First, we monitored the phosphorylation of nuclear factor kappa-light-chain-enhancer of activated B cells (NF-κB) and nuclear factor erythroid 2-related factor 2 (Nrf2), two transcription factors that play key roles in protective and inflammatory responses related to oxidative stress^31,32^. The phosphorylation of NF-κB, and to a lesser extent Nrf2, was increased in patient fibroblasts compared with controls (Fig. 2A, B). NF-κB activation was confirmed by the reduced levels of IκBα and the increased presence of phosphorylated NF-κB p65 in the nucleus of patient cells (Fig. 2C, D). This supported our hypothesis that oxidative stress is involved in MSS. Thus, we monitored the expression levels and functionality of the SOD/CAT system. Gene expression analysis by quantitative polymerase chain reaction (qPCR) of the two intracellular human SOD isoforms revealed a strong downregulation of SOD2 while SOD1 was minimally affected (Fig. 3A, B). In contrast, western blot of SOD1, and SOD2 revealed an upregulation in both isoforms, although only the increase in SOD2 was significant (Fig. 3C, D). To demonstrate that upregulation of SOD proteins corresponds to an increase in their function, we measured the enzymatic activity of SODs. Total SOD and SOD2 activity was found to be increased in patient-derived fibroblasts compared with control fibroblasts, while SOD1 activity, although slightly increased, varied non-significantly (Fig. 3E–G). The discrepancy between mRNA and protein levels of SOD is probably due to negative feedback mechanisms that sense high SOD activity. The complex relationship between NF-kB activation, ROS and SOD expression is analysed in the discussion.

**Fig. 4:**
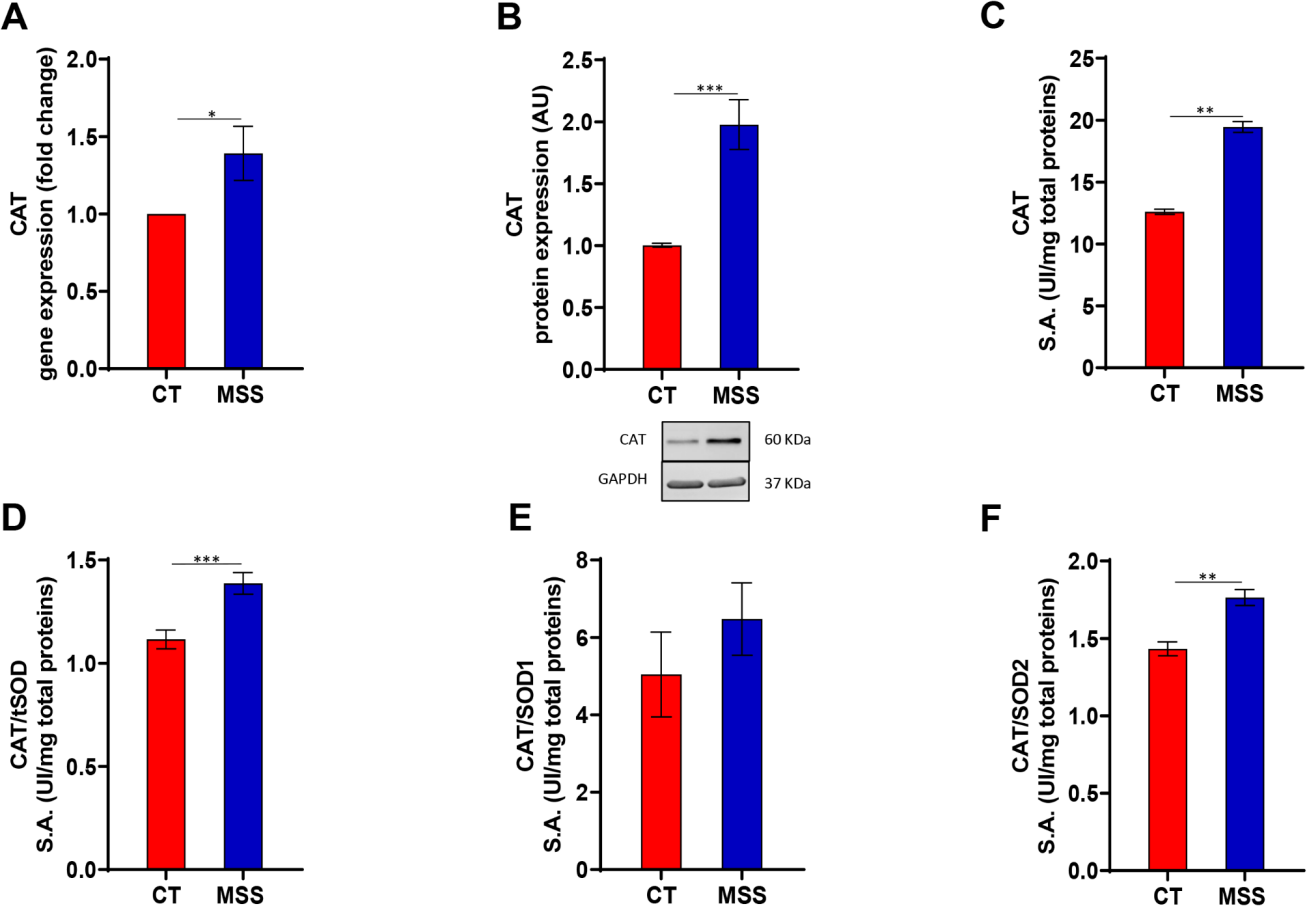
Activation of the antioxidant response and scavenging efficiency of CAT/SOD system in MSS fibroblasts. (**A**,**B**) Gene expression and protein expression of CAT. (**C**) Enzymatic activity of CAT in controls (CT) and in patient-derived fibroblasts (MSS) shows a significant increase in MSS cells. (**D**–**F**) Measurement of scavenging efficiency of CAT/SOD system using ratios between the enzymes. Statistical analysis was performed using an unpaired *t*-test with Welch’s correction (*P < 0.05, **P < 0.01, ***P < 0.001).

SODs are the main enzymes that detoxify ROS and form hydrogen peroxide, which in turn is converted to water by CAT; therefore, CAT activity and expression were evaluated in these cells. CAT activity (EC 1.11.1.6), assessed by measuring the disappearance of hydrogen peroxide at 240 nm^33^, was found to be increased in patient fibroblasts, as was the amount of the enzyme (Fig. 4A–C). CAT/SOD ratios are a measure of scavenging efficiency. An increase of CAT/SOD ratio indicates proper functioning of the cell’s detoxification system, while a decrease in it means that cellular antioxidant defences are compromised^34^. The ratio of CAT/tSOD and CAT/SOD2 activities are significantly increased in MSS compared to controls (Fig. 4D, F). The CAT/SOD1 ratio also appears to be higher, although the difference is not significant, in patient cells compared with controls (Fig. 4E). These results indicate that the patient fibroblasts put in place adaptive mechanisms based on CAT/SOD probably to counteract ROS generated by the ER stress response.

### The antioxidant defences glutathione peroxidase and reductase in MSS fibroblasts

**Fig. 5:**
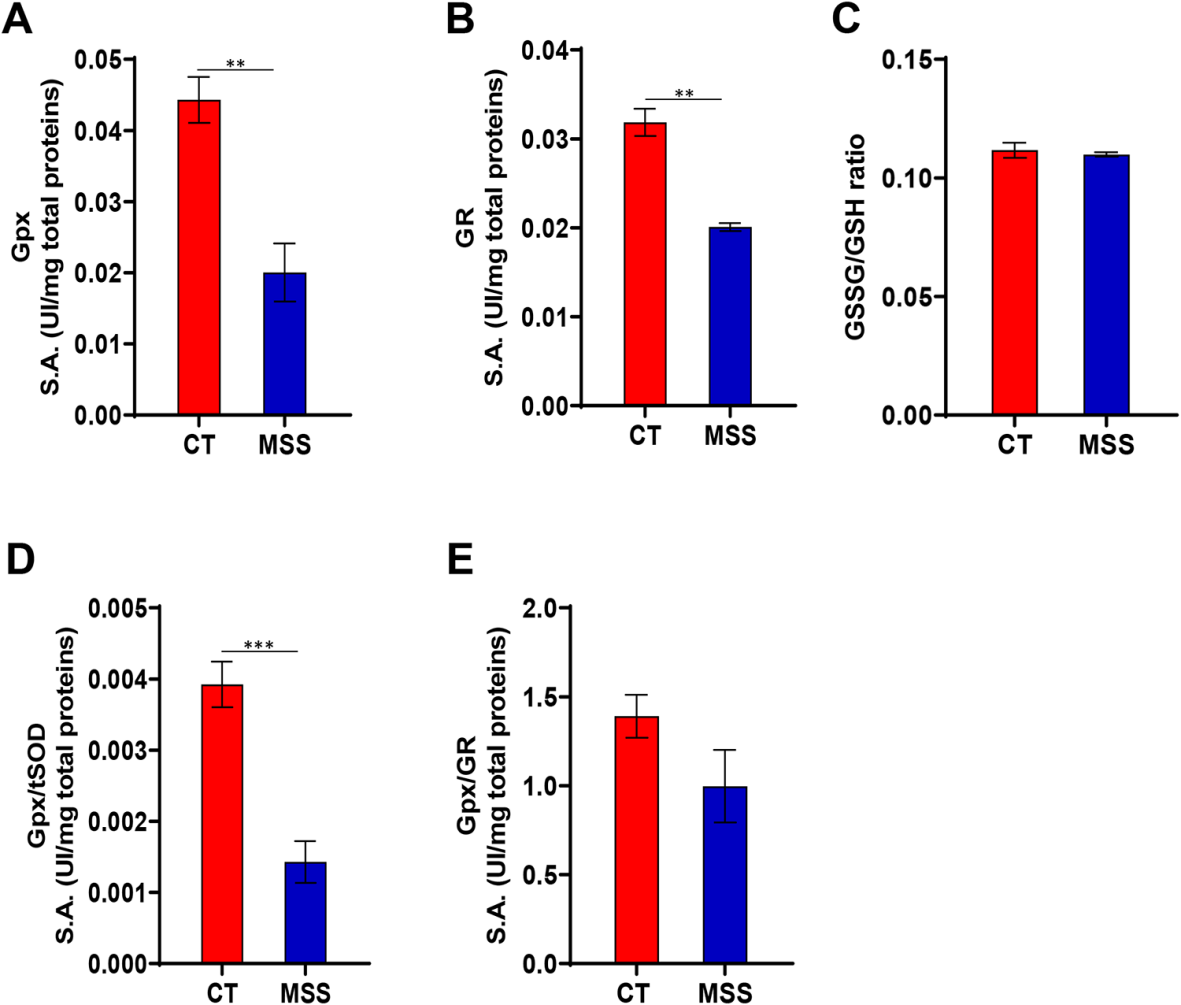
Evaluation of the activation of the detoxification system GPx/GR and redox balance in MSS fibroblasts. (**A**,**B**) GPx and GR activity show a decrease in patient-derived fibroblasts (MSS) while the ratio between oxidized and reduced glutathione (**C**) show no differences between controls (CT) and MSS fibroblast. (**D**,**E**) Measurement of scavenging efficiency of GPx/tSOD and GPx/GR system using ratios between the enzymes highlight an inefficiency of this system. Statistical analysis was performed using an unpaired *t*-test with Welch’s correction (**P < 0.01, ***P < 0.001).

Because GPx is the main enzyme that converts hydrogen peroxide to water, we also evaluated its activity in patient fibroblasts. Unexpectedly, GPx activity, measured by monitoring reduced nicotinamide adenine dinucleotide phosphate (NADPH) consumption at 340 nM (see “Methods”), was significantly decreased (Fig. 5A).

To reduce hydrogen peroxide into water, GPx uses the reduced glutathione (GSH), which is oxidized to glutathione disulfide (GSSG). Then the oxidized glutathione GSSG is reduced to GSH by GR to replenish antioxidant resources in the cell. Also GR activity, in line with GPx, was reduced in the patient fibroblasts (Fig. 5B). These data suggested a possible effect on the redox balance of glutathione and thus a reduction in its scavenging activity. The ratio of GSSG to GSH increased slightly due to a non-significant increase in the oxidised fraction in the patient fibroblasts (Fig. 5C).

Furthermore, to fully assess the capacity of cellular antioxidant defences, we also determined GPx/tSOD and GPx/GR ratios. In the patient fibroblasts, the GPx/tSOD and GPx/GR ratios decreased compared to controls (Fig. 5D, E), indicating that all the hydrogen peroxide produced by SOD is probably not reduced by GPx, yet the GPx/GR system can maintain the correct glutathione balance in cells lacking Sil1.

### Pro-oxidant molecules are kept at low levels in the fibroblasts of the patient

Because of the peculiar regulation undergone by cellular antioxidant defences, we decided to assess intracellular ROS levels (see “Methods”). No differences of ROS levels were revealed between patients and control fibroblasts (Supplementary Fig. 1A). We also evaluated the ability of cells to handle excessive ROS production by measuring ROS levels in cell treated with glucose oxidase^35^, an enzyme that generates hydrogen peroxide as a by-product of glucose oxidation. As expected, glucose oxidase increased ROS levels in both cell lines compared to basal conditions, but in the patient fibroblasts ROS levels were lower than in the controls after glucose oxidase stimulation (Supplementary Fig. 1B), suggesting that their antioxidant defences are very efficient or even overactive. To further support this result, we measured the basal levels of hydrogen peroxide in the cell culture medium. Remarkably, the hydrogen peroxide in the culture medium of the patient fibroblasts was half that of the control cells (Supplementary Fig. 1C).

### Biomarkers of oxidative damage are unchanged or reduced in patients fibroblasts

**Fig. 6:**
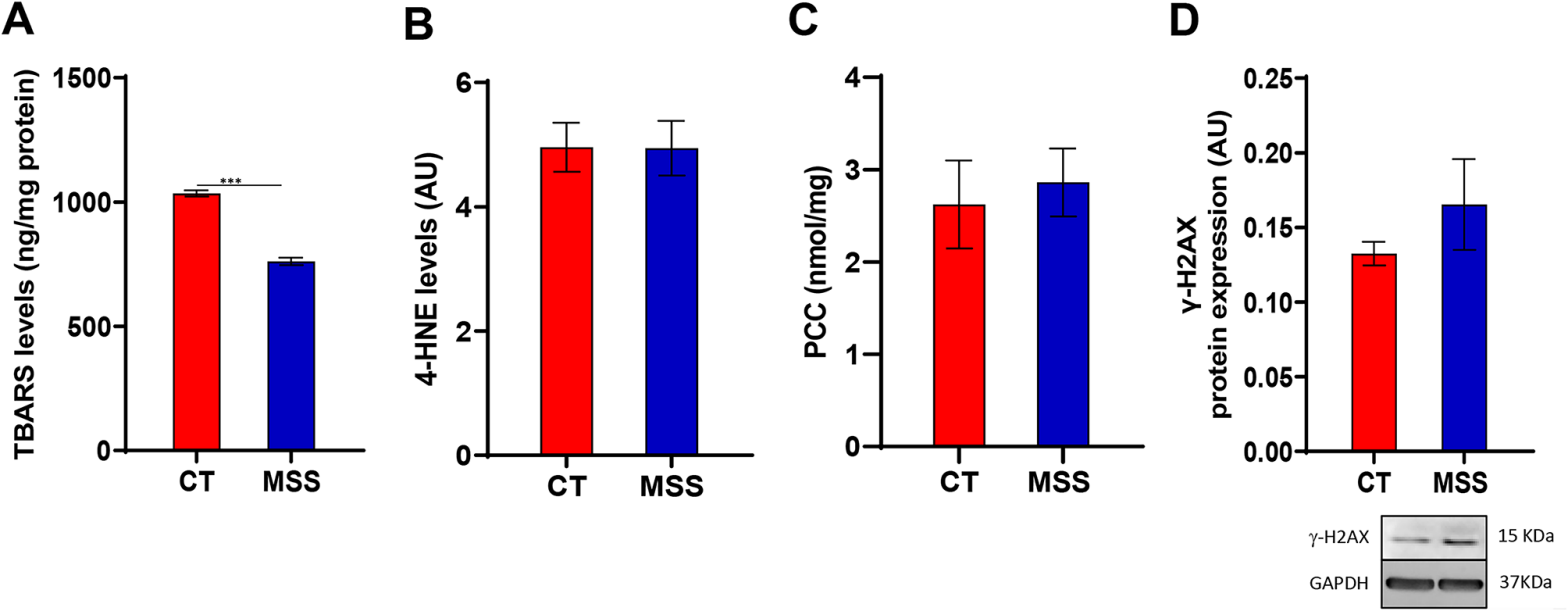
Measurement of oxidative stress-related damage marker in MSS fibroblasts. (**A**,**B**) TBARS and 4-HNE levels in CT and patient-derived fibroblasts (MSS) show a decrease for the first one while no significant differences were observed for the second one. (**C**) Protein Carbonyl Content (PCC) quantification in CT and MSS fibroblasts shows no differences. (**D**) The measurement of ROS-related DNA damage highlight a non-significant upward trend. Statistical analysis was performed using an unpaired *t*-test with Welch’s correction (***P < 0.001).

Since the antioxidant defences of the patient fibroblasts appear very efficient, we assessed oxidative damage by measuring MDA, produced by polyunsaturated fatty acids peroxidation, as a marker. MDA levels were measured following the reaction with thiobarbituric acid (see “Methods”). In line with enhanced antioxidant defences, we observed a decrease in MDA in the patient fibroblasts, indicative of a general reduction of lipid peroxidation (Fig. 6A). To extend these results, we also measured 4-HNE, a specific marker of ω-6 fatty acid peroxidation. 4-HNE levels were comparable in patients fibroblasts and controls (Fig. 6B). Next we evaluated the oxidative damage of proteins by measuring the PCC (see “Methods”). Carbonyl groups, both aldehydes and ketones, can be produced on the side chains of specific amino acids when they are oxidised. This analysis showed similar levels of PCC in patient and control fibroblasts indicating that in patient fibroblasts, despite the UPR and the possible generation of high levels of ROS, efficient CAT/SOD antioxidant defences prevent protein damage (Fig. 6C).

Finally, we assessed possible DNA damage, including double-strand breaks, by measuring the γ-H2AX, a phosphorylated variant of histone H2A, as a marker. Patient fibroblasts showed only a small, non-significant increase in γ-H2AX compared to control cells, confirming that the antioxidant resources of these cells are sufficient to counteract oxidative stress (Fig. 6D).

### Woozy mouse skeletal muscle confirmed the enhancement of the SOD/CAT antioxidant system and the reduction of lipid peroxidation

**Fig. 7:**
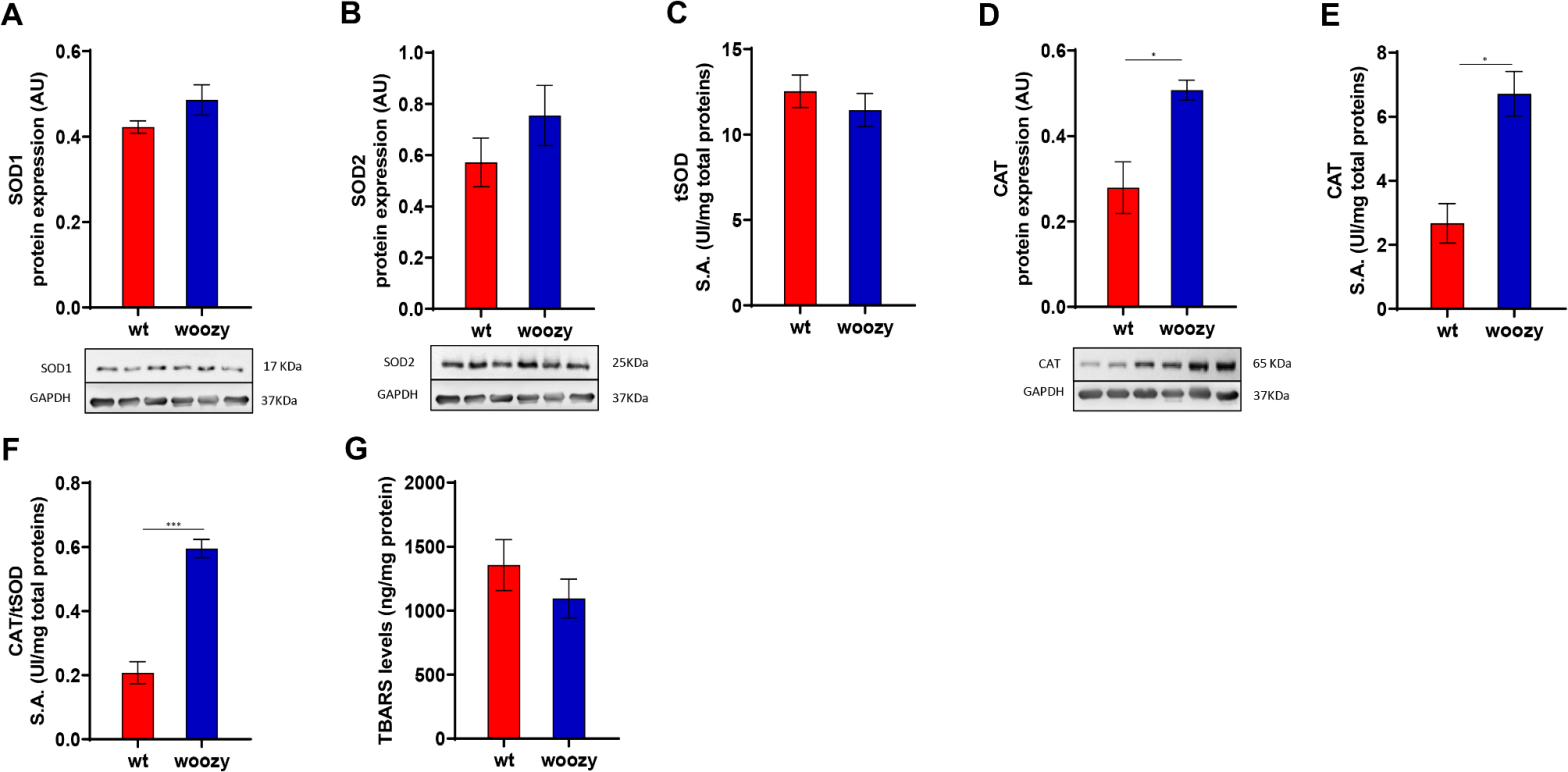
Antioxidant response in muscle tissue of woozy mice. (**A**,**B**,**D**) Protein expression level of CAT and SOD1/2 show a significant increase for CAT while an increasing trend for the last ones. (**C**,**E**) Enzymatic activity of CAT and total SOD in muscle tissues. (**F**) Measurement of scavenging efficiency of CAT/SOD system using ratios between the enzymes. (**G**) Measurement of TBARS as marker of lipid damage ROS-related shows the same trend observed for CT and MSS cells. Statistical analysis was performed using an unpaired *t*-test with Welch’s correction (*P < 0.05, ***P < 0.001).

To validate the results obtained in the fibroblasts isolated from a patient with MSS, we examined the SOD/CAT antioxidant system and the level of oxidative damage in the skeletal muscle of the woozy mouse, a representative model of MSS. Western blot analysis showed a trend toward increased expression of SOD1 and SOD2 in the quadriceps of woozy mice, although given the variability among animals, the effect did not reach significance (Fig. 7A, B). While, tSOD activity in woozy quadriceps was comparable to controls (Fig. 7C). Concerning CAT, both expression and activity was significantly augmented in the woozy mouse quadriceps (Fig. 7D, E). Putting all these together, we see that scavenging activity expressed as CAT/tSOD has increased significantly (Fig. 7F). Finally, similar to patient fibroblasts, MDA levels showed a downward trend in woozy mouse muscle compared with wild-type control animals (Fig. 7G).

These data confirm that the changes in antioxidant defences observed in patient fibroblasts is a general characteristic of MSS pathology, as it is also present in the skeletal muscle of woozy mice.

## Discussion

MSS is a neuromuscular disease triggered by the loss of the ER co-chaperone Sil1. The lack of Sil1 impairs protein folding, leads to UPR and indirectly alters mitochondrial morphology^23,26–28,36,37^. The UPR can increase ROS levels as a by-product of oxidative folding in the ER. Also, dysfunctional mitochondria can produce more ROS than healthy properly functioning ones. Thus, elevated levels of ROS and mitochondrial issues are both linked with oxidative stress and have been implicated in neurogenerative diseases including Alzheimer’s, Parkinson’s, Huntington’s, ALS, and ataxias^13,15–17,38−40^.

In the present study, we analysed energy production in fibroblasts isolated from a patient affected by MSS to understand what fraction of ATP is generated by the glycolytic pathway and what by the mitochondria. We also analysed the rate of ATP generation by these fibroblasts to confirm the higher demand generally associated with UPR^6,7^. As expected, mitochondria produced the most ATP compared to glycolysis in both patient fibroblasts and controls. However, fibroblasts isolated from the patient exhibited a lower rate of mitochondrial ATP production and a reduced maximal mitochondrial respiration capacity compared to controls.

These data may appear at odds with our previous study that proposed an activation of amino acid catabolism, beta-oxidation, and increased Krebs cycle in patients cells to generate a high amount of energy^26^. We would like to emphasize that, although both studies were conducted on the same cells, for some aspects cannot be directly compared. In our previous study, we performed a proteomic analysis, whereas in the current study, we used biochemical assays to measure enzyme activities and functional outcomes. Mitochondrial impairment, observed in the current study, may have triggered an adaptive response that increases the protein levels of enzymes involved in energy metabolism without leading to enhanced oxidative metabolism.

Indeed, this manuscript provides, for the first time, clear evidence of mitochondrial dysfunction in MSS, consistent with prior studies reporting mitochondrial abnormalities^28,36,37,41,42^. Consistent with these findings, our previous proteomic analysis identified 99 mitochondrial proteins with altered expression, encompassing both matrix and membrane proteins^26^. We also observed an upregulation of mitofusin-2, a key regulator of mitochondrial fusion and mitochondria-ER contact site organization (Supplementary Fig. 2). Thus, based on the prior data and the findings from the current study, we propose that mitochondrial dysfunction is a key event in the neurodegeneration associated with MSS. Note that a prominent role of mitochondrial impairment in neurodegeneration and ataxia is well recognized^40^.

In our previous omics studies we revealed an upregulation of CAT and SODs^26,30^, thus to investigate the possible involvement of oxidative stress in MSS, we first evaluated the amounts of the phosphorylated forms of the transcription factors Nrf2 and NF-κB. Nrf2, by binding to the AU-rich element sequence (ARE) of specific genes, including SOD2, CAT, GST GPx, is an important controller of the oxidative stress response^43^. NF-κB is mainly involved in transcription of genes related to inflammation, apoptosis and oxidative stress processes, including cytokines, growth factors and GPx^32,43,44^. Our data showed that, in the MSS samples, there was no significant difference in the phosphorylated form of Nrf2, whereas a significant increase in the phosphorylation of NF-κB was observed. This transcription factor is sensitive to the cellular redox state and might promote SOD2 gene transcription through the p65/50 complex^45^. However, despite an increase in SOD2 activity, we observed a reduced expression of SOD2 mRNA, suggesting that this regulatory pathway is not involved. On the other hand, SOD2 itself has the potential to activate NF-κB signalling pathways^46^. NF-kB activation by SOD2 could help maintain cellular homeostasis by promoting autophagy, a key event in MSS^26,37^ and in oxidative stress^32,47^. As mentioned, unlike its mRNA, SOD2 protein and enzyme activity were significantly increased in patient cells compared with controls. One possible explanation for these apparently conflicting results is that SOD2 protein levels are upregulated through post-translational mechanisms, such as reduced activity of the ubiquitinating enzyme USP36^48^, and that subsequently a feedback adaptation reduces mRNA levels (e.g., via miRNA).

Furthermore, CAT gene and protein expression, along with enzymatic activity, were found to be upregulated in fibroblasts from MSS patients compared to controls. Increased CAT activity was reported in studies related to ataxia and Nijmegen syndrome (NBS) syndrome^49,50^. An increase in CAT activity was also reported after exposure to exogenous hydrogen peroxide or other oxidants as inducers of ROS^51–53^.

Overall, our findings suggest an activation of the redox response, as evidenced by the significantly increased CAT/SOD1, CAT/SOD2, and CAT/tSOD ratios in patients compared to controls. We confirmed the activation of antioxidant response mechanisms in MSS in the woozy mouse, a representative animal model of this syndrome^23,54^. Indeed, in the quadriceps of these mice, the CAT/tSOD ratio was significantly increased and lipid peroxidation reduced.

In line with a strong activation of antioxidant defences, patient-derived fibroblasts after a glucose oxidase insult showed an increased ability to defend themselves against ROS and detoxify them. The central role of the CAT/SOD system in the detoxification of ROS^49^ even after the exogenous insult with glucose oxidase was previously reported^49^.

We further analysed the cellular redox status by evaluating the ratio between GSSG/GSH. Glutathione is not only an antioxidant molecule in itself but also acts as a cofactor for the reaction catalysed by the GR-GPx enzyme system^55^. The GSSG/GSH ratio in patient fibroblasts showed a little shift of the cellular redox balance towards a more oxidized state suggesting a lower efficiency of the GPx/GR scavenging system^34^. Indeed, the activities of GPx and GR were significantly decreased in patient cells. Similar observations come from studies showing increased susceptibility to oxidative stress in ataxia sufferers with reduced GPx levels^56^. A reduction in GR activity has been described in rat muscles under hyperoxic conditions^34^. The reduced GSH level and the shift towards an oxidised state could underlie the reduced enzyme activity observed. This leads to a decrease in scavenging efficiency, as demonstrated by the GPx/tSOD, GPx/SOD1 and GPx/SOD2 ratios^34^.

Given the conflicting results between the increase in CAT/SOD and the downregulation of GPx/GR, we also assessed the total detoxifying capacity by quantifying intracellular ROS and hydrogen peroxide in the culture medium. Both oxidants were significantly lower in the patient fibroblasts, indicating that the modified setting of detoxifying enzymes results in a very efficient system. The reduced activities of GPx and GR may be associated with the low hydrogen peroxide levels resulting from the increased activity of CAT^57^.

This scenario prompted us to evaluate ROS-dependent damage markers, including MDA, PCC and 4-HNE. Although no differences were found for PCC and 4-HNE, the lipid peroxidation damage marker, MDA, was lower in the patient cells and in the quadriceps of woozy mice. A decrease in MDA, albeit not significant, was previously reported in subjects with ataxia telangiectasia^58^. We have previously reported increased expression of aldehyde dehydrogenases in MSS, including ALDH1B1, ALDH3A2, ALDH6A1 and ALDH7A1, which could contribute to the removal of lipid peroxidation by-products and thus explain the low levels of MDA and 4-HNE^26,59^.

In conclusion, although MSS leads to the generation of a potential oxidative intracellular environment, the cellular response can cope with it efficiently through upregulation of the CAT/SOD system.

## Materials and methods

### Antibodies

Abcam (Cambridge, UK) provided the following primary antibodies: anti-SOD1 (ab16831; dil.1:1000), anti-SOD2 (ab86087; dil.1:1000), anti-CAT (ab16731; dil. 1:2000), anti-4HNE (ab46545; dil. 1:3000), anti-p-Nrf2 (cat. ab76026, dil. 1:5000), anti-p-NFkB (cat. sc-136548; dil. 1:500). Cell Signaling Technology (Danvers, Massachusetts, USA) provided the anti-GAPDH (cat. D16H1; dil. 1:10000). The HRP-conjugated goat anti-rabbit IgG secondary antibody (cat. 7074 S; dil. 1:2000) was purchased from Cell Signaling Technology. The peroxidase-conjugated anti-mouse secondary antibody (A9044; dil. 1:1000) was purchased from Sigma-Aldrich (Milan, Italy).

### Mouse handling and ethics statement

Woozy mice (CXB5/By-Sil1wz/J) were sourced from The Jackson Laboratory (Stock No. 003777) and kept under controlled humidity and temperature conditions. They had unrestricted access to a standard diet. The mouse colony was maintained through heterozygous breeding, and genotyping was conducted using PCR. All procedures involving mice were performed according to the relevant guidelines and regulation (EEC Council 2010/63/EU and Italian D.Lgs. 26/2014). The protocol was approved by the Institutional Animal Care and Use Committee of the Italian Ministry of Health (authorization n° 596/2021-PR).The study was carried out in compliance with the ARRIVE guidelines (https://arriveguidelines.org).

### Culture of primary human skin fibroblasts

NDHF (Promo Cell #FB60C12350) supplied by Carlo Erba was used as the control cell line and compared with primary dermal fibroblast from a young patient with MSS, supplied by Telethon Network of Genetic Biobanks—TNGB^60^. Cells were cultured in Dulbecco’s modified Eagle’s medium + GlutaMAX (GIBCO, 10566-016), supplemented with 10% of Foetal Bovine Serum (FBS) and 1% penicillin/streptomycin PenStrep (Euroclone, ECB3001D). Cells were maintained at 37 °C, 5% CO_2_, and detached by Trypsin-EDTA 0.5% (Euroclone, ECB3053D).

#### Preparation of cell extract for evaluation of enzyme activities

Controls and patient fibroblasts (6 × 10^6^ cells/ml) were lysed in 100 mM phosphate buffer (pH 7), containing 1.5 mM dithiotreitol (DTT) and 1 mM EDTA for total glutathione peroxidase and 100mM phosphate buffer (pH 7), containing 0.1% (v/v) Triton X-100 for total SOD, SOD2 and CAT. The samples underwent three freeze/thaw cycles in liquid nitrogen and then were homogenized and centrifuged at 16,000×*g* for 30 min at 4 °C. Supernatants were used for spectrophotometric measurement of total protein concentration by using the BCA protein assay kit and albumin as standard (cat. 23225; Thermo Fisher Scientific, Milan, Italy), and for evaluation of all enzyme activities. All spectrophotometric readings were taken in triplicate using a Lambda spectrophotometer25 (PerkinElmer, Inc., Waltham, MA, USA).

#### Measurement of SOD activity

Total SOD (EC 1.15.1.1) activity was assayed in 50 mM sodium carbonate buffer (pH 10.2), containing 25 mM EDTA and 0.1 M epinephrine bitartrate (cat. E4375, Sigma-Aldrich, Milan, Italy). We monitored SOD’s ability to inhibit the auto-oxidation of epinephrine at 480 nm, according to Sun and Zigman 1978^61^. We defined one unit of enzyme the amount required to halve the rate of epinephrine autoxidation at 30 °C. After tSOD activity evaluation, we inactivated SOD1 using 1 mM KCN (cat. 207810, Sigma-Aldrich, Milan, Italy)^62^ and we measured the SOD2 activity. SOD1 activity was calculated as tSOD-SOD2.

#### Measurement of CAT activity

CAT activity (EC 1.11.1.6) was assayed following Aebi’s protocol^34^. We recorded the disappearance of 10 mM hydrogen peroxide at 240 nm and 25 °C (cat. 21,676-3; Sigma-Aldrich). One unit was defined as 1 µmol of H_2_O_2_ reduced/min.

#### Measurement of total glutathione peroxidase (tGPx) activity

Quantification of glutathione peroxidase activity was measured using a buffer containing 50mM KH_2_PO_4_, 1 mM EDTA, 1.5 mM sodium azide (NaN_3_, cat.S2002, Sigma Aldrich, Milan, Italy) (pH 7) with 1.3 mM GSH (4 mg/10 mL buffer, cat. G4251, Sigma Aldrich, Milan, Italy), GR (4 U/10 mL buffer, cat.G3664, Sigma Aldrich, Milan, Italy), NADPH (cat. N7505, Sigma Aldrich, Milan, Italy) dissolved in 5.7 mM ddH_2_O (double-distilled H_2_O) and adding H_2_O_2_ (11.5 µL 30% H_2_O_2_ + 15 mL ddH_2_O). The decrease in absorbance was measured by spectrophotometric reading at 340 nm at 25 °C after the addition of H_2_O_2_. The specific activity is given as nm of GSH oxidized per minute per mg of protein^63^.

#### Measurement of GR activity

The activity of GR (GSSG-Rx; EC 1.6.4.27) was measured using a buffer containing 50 mM potassium phosphate pH 7.4, 1 mM EDTA, 1 mM GSSG (cat. G4376, Sigma Aldrich, Milan, Italy) and 0.16 mM NADPH. We followed the oxidation of NADPH at 25 °C using a spectrophotometer at 340 nm. One unit was defined as 1 µole of NADPH oxidized/min^64^.

### Measurement of reduced and oxidized glutathione

Total GSH and GSSG levels were determined by using a glutathione assay kit (cat. 703002, Cayman Chemical)^65^. Briefly, CT and MSS fibroblast were lysed in MES buffer provided by the kit (1.5 × 10^7^ cells/mL), and centrifuged at 10,000×*g* for 15 min, at 4 °C. Cell lysates were deproteinized with 5% (w/v) metaphosphoric acid (cat. 239275, Sigma-Aldrich, Milan, Italy) and centrifuged at 4000×*g* for 5 min, as recommended by the supplier. To evaluate the abundance of GSSG, GSH in the samples was first derivatized with 2-vinylpyridine (cat. 132292, Sigma-Aldrich, Milan, Italy). Protein-free samples (50 µL) and the assay cocktail (150 µL) were loaded in a 96-well plate, in triplicates. Absorbance at 405 nm was followed for 30 min, with 5 min intervals, by using a Victor3 microplate reader (PerkinElmer, Waltham, MA, USA). tGSH and GSSG concentrations of experimental samples were interpolated on calibration curves that were obtained from reactions containing either pure GSH or pure GSSG standards (0–16 µM tGSH or 0–8 µM GSSG).

### Measurement of MDA

MDA levels were measured by thiobarbituric acid reactive substances (TBARS) according to the protocol described by Yagi^66^, using the TBARS Assay kit (cat. 10009055, Cayman Chemical, Ann Arbor, MI, USA). Briefly, the cells were extracted in 37.5 µL PBS and the resulting samples were mixed with 1 volume of SDS. Then, color reagent was added to each sample in triplicates. The reaction mixtures were incubated for 1 h in boiling water and centrifuged at 1600×*g* for 10 min at 4 °C. Supernatants were read at 532 nm by a MultiSkan Go microplate spectrophotometer (ThermoFisher Scientific, Waltham, MA, USA). TBARS concentrations of unknown samples were interpolated on a linear calibration curve that was obtained from pure MDA-containing reactions (0–50 µM).

### Measurement of hydrogen peroxide

Fibroblasts (1 × 10^6^ cells/ml) were detached and centrifuged at 300×*g* for 5 min, the medium was collected and centrifuged at 1000×*g* for 30 min and then deproteinized according to the hydrogen peroxide assay kit manufacturer’s instructions. (Abcam, cat. Ab102500). Briefly, cold perchloric acid (PCA) 4 M was added to the samples that were kept in ice for 5 min and then centrifuged. After, cold KOH 2 M (34%v/v) was used to precipitate the excess of PCA and samples were centrifuged at 13,000×*g* at 4 °C for 15 min to collect deproteinized medium. The H_2_O_2_ concentration of unknown samples was interpolated on a calibration curve obtained from standard H_2_O_2_ (0–5 nm/well). The reaction mix, 50 µl of standards/samples and 50 µl of the colorimetric mixture, was incubated at room temperature for 10 min and then the H_2_O_2_ content was measured at 570 nm by using a MultiSkan Go microplate spectrophotometer.

### Measurement of protein carbonyl content

Fibroblast cell pellets (1,500,000 cells) were extracted in 50 mM KH_2_PO4 extraction buffer, pH 6.7 with 1mM EDTA. The samples were frozen and thawed in liquid nitrogen 3 times, centrifuged at 16,000×*g* for 30′ at 4 °C and the supernatant stored at − 80 °C. A 10% (w/v) streptomycin sulphate (SS, cat. Sc-202821, Santa Cruz Biotechnology) solution in 50 mM pH 7.2 KH_2_PO_4_ buffer was used to remove interfering nucleic acids. The samples were then processed according to the protein carbonyl colorimetric assay kit instructions based on 2,4-dinitrophenylhydrazine (DNPH) (Cayman Chemical Kit, cat. 10005020). The values were obtained by reading the absorbance at 280 nm and interpolating the data in the calibration line obtained by preparing 3 points of BSA standard (Sigma Aldrich, cat. A-6003).

### Measurement of cell ROS levels

Fibroblasts were seeded at a density of 2000 cells/well in a 96-well plate to assess ROS levels both at baseline and stimulated by glucose oxidase using the DCFDA (cat.ab113851). The day after seeding, the cells were treated with 0.5 U/ml of glucose oxidase for 30 min. After 30 min the cells were washed with buffer and stained with DCFDA according to manufacturer’s instruction. The samples were read by using Victor 3 microplate reader (excitation 495/emission 535).

### Total protein extraction and Western blot analysis

Whole-cell CT and MSS cell were lysed in RIPA buffer (cat. R0278, Sigma-Aldrich, Milan, Italy), supplemented with protease and phosphatase I and III inhibitors (cat. P8340, Sigma-Aldrich, Milan, Italy) (cat. P2850 and P5726from Sigma-Aldrich, Milan, Italy). After centrifugation at 16,000×*g* for 30 min at 4 °C, supernatants were assayed for total protein content, by using the BCA Protein Assay Kit and bovine serum albumin (BSA) as the standard (cat. PR23225, Thermo Fisher Scientific, Milan, Italy). Denatured samples (10–20 µg) were run in triplicates on polyacrylamide gels (12%), and then transferred onto nitrocellulose membranes by electrophoretic transfer. Non-specific binding sites were blocked at room temperature for 1 h with 5% (w/v) Blotting-Grade Blocker (cat. 170-6404, Bio-Rad Laboratories, Hercules, CA, USA), in Tris-buffer saline containing 0.05% (v/v) Tween-20 (cat. P5927, Sigma-Aldrich, Milan, Italy) (TBS-T). We diluted primary antibody in TBS-T and we incubated membranes overnight. The next day, membranes were washed in TBS-T and incubated, with the peroxidase-conjugated secondary antibody for 2 h. The images were revealed, acquired and analysed by using Enhanced Chemi-luminescent Substrate Kit (cat. EMP001005, Euroclone, Milan, Italy), AllianceLD2 hardware (UVItec Limited, Cambridge, UK), and Total Lab TL120 software (TotalLab, Newcastle upon Tyne, UK). GAPDH was used as the loading control for data normalization.

### RNA extraction and real-time quantitative relative PCR

RNA was extracted from fibroblasts by using Ribospin kit (cat. 304-150), and the possible contaminant DNA was degraded by Riboclear plus (cat. 313-150) (all from GeneAll Biotechnology, Seoul, South Korea), following the supplier’s recommendations. The resulting RNA was used to obtain cDNA via reverse transcription (cat. NP100041, OriGene Technologies, Rockville, MD, USA). The cDNA (dil. 1:100) was used for PCR in an Applied Biosystems 7300 system (ThermoFisher Scientific, Rockford, IL, USA), using primers human-specific (300 nM/reaction) and SensiFAST SYBR MasterMix (cat. BIO-92005, Bioline). The amplification reaction was as follows: 1° step 2 min at 95 °C, 2° step 5 s at 95 °C, then 40 cycles of 30 s at 60 °C, 3° step 15 s at 95 °C, 1 h at 60 °C, 15 s at 95 °C and 15 s 60 °C (melting curve). Gene expression was calculated by using the 2−∆∆Ct method, using 18 S as the reference RNA and control sample as the calibrator (Livak e Schmittgen, 2001). The assays were performed with three replicates.

Primers: CAT Fw 5′-TTGCCACAGGAAAGTACCCC-3′, Rv 5′-ACCAACTGGGATGAG AGGGT-3′; SOD2 Fw 5′-AGGCTCAGGTTGGGGTTGGCT-3′, Rv 5′-GCGTGCTCCCACACATCAATCCC-3′; SOD1 Fw 5′-AGGCATGTTGGAGACTTGGG-3′ Rv 5′-CCACAAGCCAAACGA CTTCC-3′; 18 S Fw 5′-ACTCAACACGGGAAACCTCA-3′ Rv 5′-AACCAGACAAATCGCTCCAC-3′.

### Seahorse XF real-time ATP rate assay

The Seahorse XF real-Time ATP Rate Assay and Cell Mito Stress Test (Agilent, cat. 103592-100 and 103015-100) were used according to the manufacturer’s instructions to assess the level of total ATP, the fraction from the glycolytic and mitochondrial pathways by measuring oxygen consumption rate (OCR), proton efflux rate (PER) and mitochondrial function. Briefly, the day before the assay, control fibroblasts (CT) and patient-derived fibroblasts cells were seeded in a 96-well Seahorse utility plate at a seeding density of 20,000 cells/well and cultured overnight to allow cell adhesion. Prior to the assay, cells were washed, and the medium was replaced with Seahorse XF DMEM supplemented with 20 mM glucose, 2 mM l-glutamine and 1 mM sodium pyruvate (cat. 103680-100). For both the assays Oligomycin and Rotenone/Antimycin were used at a final concentration per well of 1.5 µM and 0.5 µM, respectively. FCCP was used at a final concentration per well of 0.5 µM for the Mito Stress test. Data were normalized to the number of cells.

### Fluorescence immunostaining and confocal microscopy

Cells were plated on coverslips at 70% density. The day after, the cells were fixed with 4% PFA (Electron Microscopy Science) for 10 min at room temperature. Cells were subsequently incubated for non-specific site blocking and permeabilization in blocking solution [0.05% saponin, 0.5% bovine serum albumin, and 50 mM NH4Cl in PBS] for 40 min at room temperature. Primary antibody incubation was performed overnight at + 4 °C. (p-NFkB Santacruz #33020 1:25; Mfn2 Cell Signalling #9482 1:100). The day after, fluorescent Alexa Fluor-conjugated anti-IgG secondary antibody was incubated for 40 min at room temperature and DAPI was used as nuclei counterstain. Fluorescence was detected using a LSM800 Zeiss confocal microscope and images were post-processed and analyzed using Fiji software.

### Statistic analysis

Microsoft Excel 2007, GraphPad Prism 9 and Total Lab TL120 software packages were used for data processing and visualization. Statistical analysis was performed with Student’s *t*-test (hypothesis test statistic) with Welch’s correction. The differences were considered statistically significant at *p* < 0.05.

## Electronic supplementary material

Below is the link to the electronic supplementary material.

Supplementary Material 1

Supplementary Material 2

## Data Availability

The data and materials in this article are available with the agreement of corresponding authors.
