# Supplementary material for: Selective activation of antioxidant resources and energy deficiency in Marinesco–Sjögren syndrome fibroblasts as an adaptive biological response to Sil1 loss
Source: Sci Rep. 2025 Apr 11;15:12510. doi: 10.1038/s41598-025-96467-9 (PMC11992280; doi:10.1038/s41598-025-96467-9)
Supplement: Supplementary file 1 — Supplementary Material 1 [file 41598_2025_96467_MOESM1_ESM.pdf]

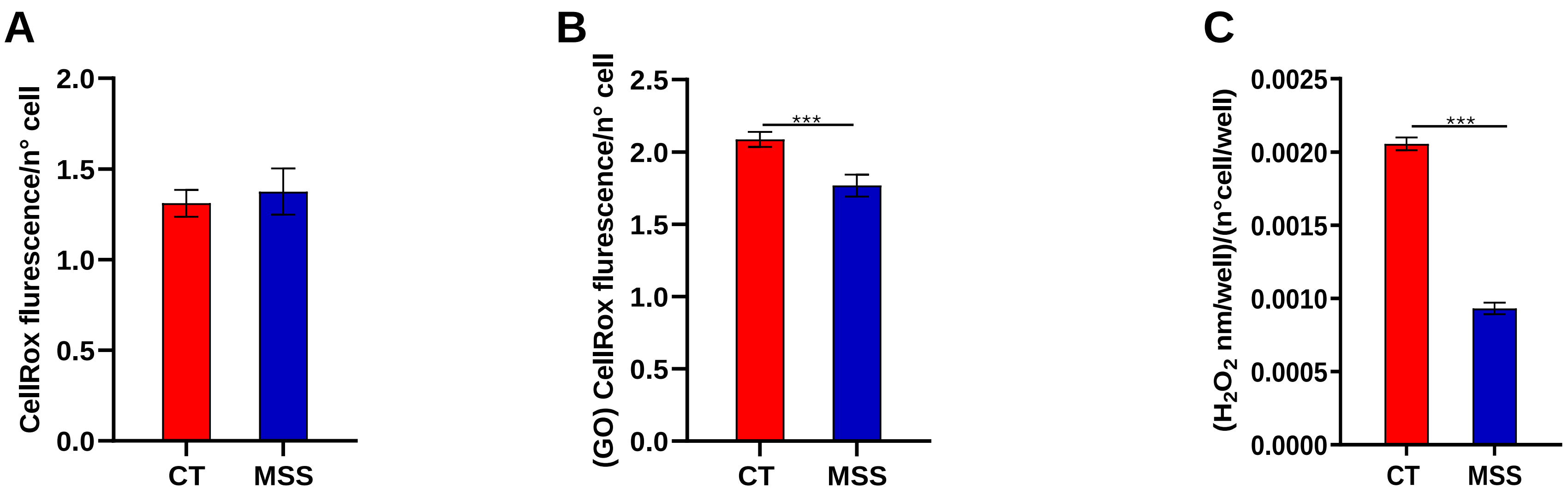

Supplementary figure 1. Assessment of Reactive Oxygen Species levels in CT e MSS cells. (A,B) The graphs show equal basal ROS levels between CT and MSS, while after stimulation with glucose oxidase lower ROS levels are evident in MSS cells, probably due to a greater responsiveness of the detoxification system (C) Measurement of hydrogen peroxide concentration in the culture medium in CT and MSS cells. Statistical analysis was performed using an unpaired t-test with Welch's correction (***P<0,001).

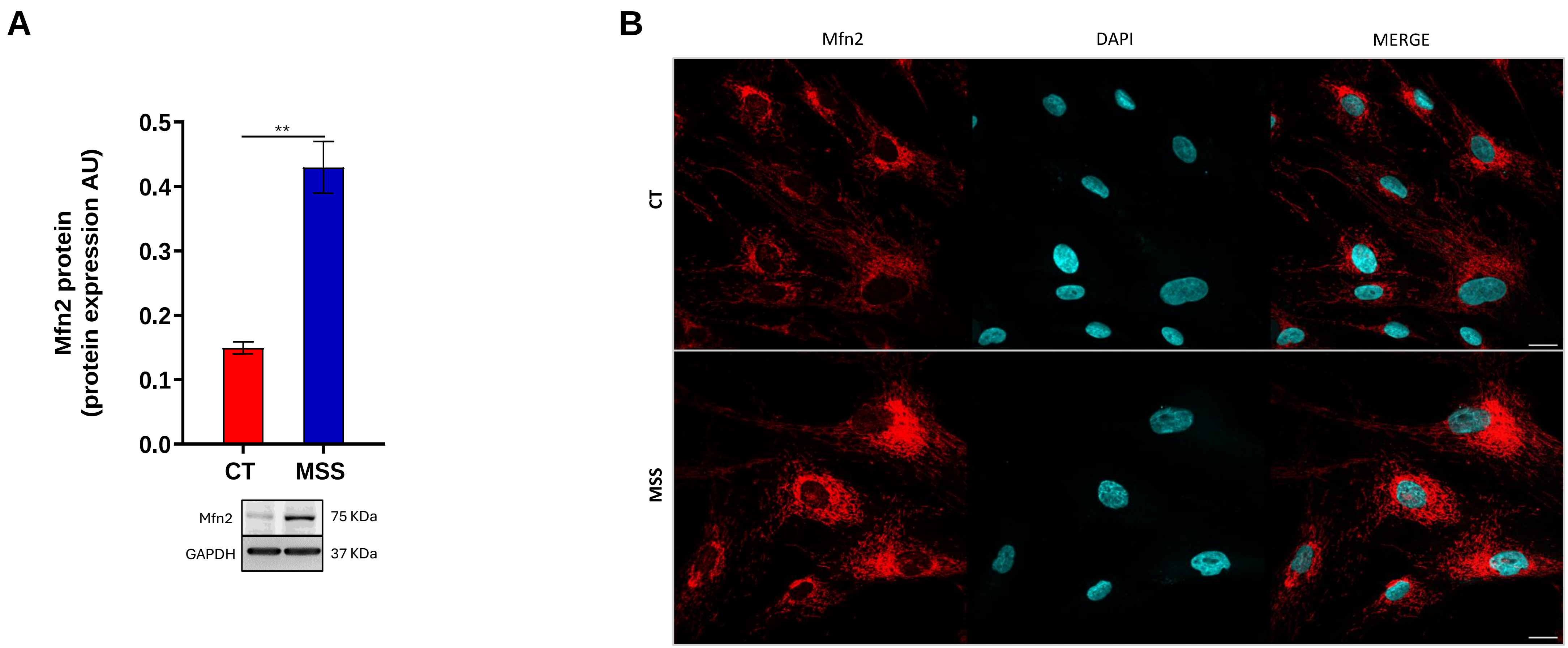

Supplementary figure 2. Patient-derived fibroblasts exhibit increased expression of Mfn2. (A) Western blot showing the expression of Mfn2 in controls (CT) and patient-derived fibroblasts (MSS). Statistical analysis was performed using an unpaired t-test with Welch's correction (**P<0,01). (B) IF analysis of Mfn2. Controls (CT) and patient cells (MSS) were plated to 70% confluency, and the following day, cells were fixed and processed for IF. Mfn2 staining is shown in red, and DAPI in blue. Merged images are also provided. Scale bar: 20 µm.
