# Supplementary material for: Selective activation of antioxidant resources and energy deficiency in Marinesco–Sjögren syndrome fibroblasts as an adaptive biological response to Sil1 loss
Source: Sci Rep. 2025 Apr 11;15:12510. doi: 10.1038/s41598-025-96467-9 (PMC11992280; doi:10.1038/s41598-025-96467-9)

SUPPLEMENTARY INFORMATION II - SOURCE DATA

Selective activation of antioxidant resources and energy deficiency in Marinesco-Sjögren syndrome fibroblasts as an adaptive biological response to Sil1 loss

Valeria Panella[1], Francesca Potenza[2,3], Carla Tatone[4], Lorenza Speranza[1], Fernanda Amicarelli[4] and Michele Sallese[2,3,*]

[1]Department of Medicine and Aging Sciences, "G. d' Annunzio" University of Chieti-Pescara, 66100 Chieti, Italy.
[2]Department of Innovative Technologies in Medicine and Dentistry, 3Center for Advanced Studies and Technology (CAST), "G. d' Annunzio" University of Chieti-Pescara, 66100 Chieti, Italy.
[4]Department of Life, Health and Environmental Sciences, University of L'Aquila, L'Aquila 67100, Italy.

*Corresponding author: Michele Sallese;
Department of Innovative Technologies in Medicine and Dentistry,
'G. d' Annunzio' University of Chieti–Pescara, Chieti, Italy
Tel: +39-0871-541427
e-mail: Michele.sallese@unich.it

Figure 2A:

NFkB phosphorylation in patient-derived fibroblasts (MSS) and controls (CT).

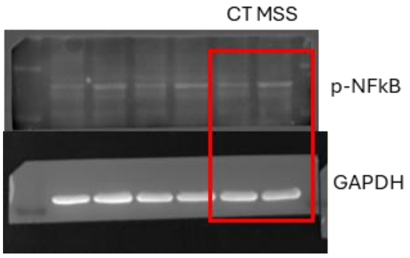

Figure 2B:

Nrf2 phosphorylation in patient-derived fibroblasts (MSS) and controls (CT).

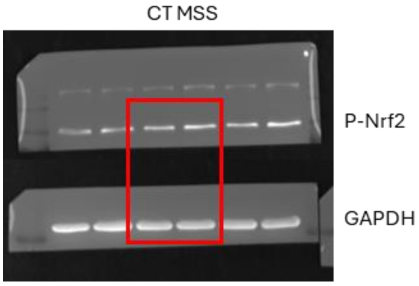

Figure 2C:

IκBα expression levels in patient-derived fibroblasts (MSS) and controls (CT).

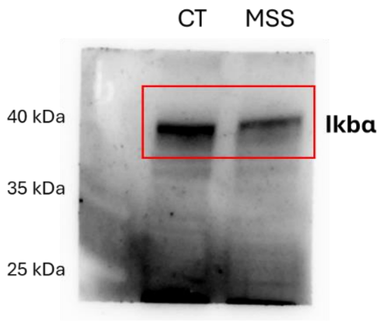

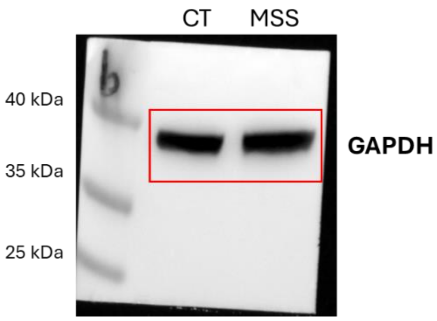

Figura 3C:

Western blot analysis of SOD1 in patient-derived fibroblasts (MSS) and controls (CT).

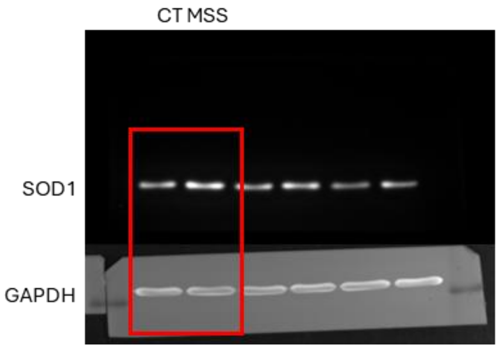

Figure 3D:

Western blot analysis of SOD2 in patient-derived fibroblasts (MSS) and controls (CT).

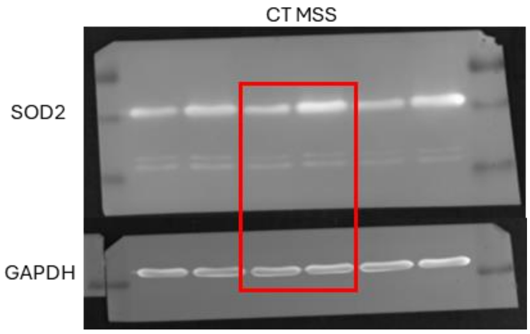

Figure 4B:

Western blot analysis of CAT in patient-derived fibroblasts (MSS) and controls (CT).

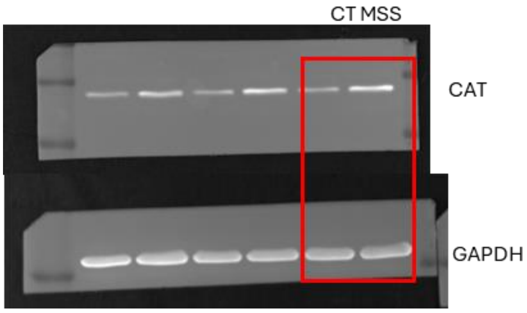

Figure 6D:

Western blot analysis of γ-H2AX in patient-derived fibroblasts (MSS) and controls (CT).

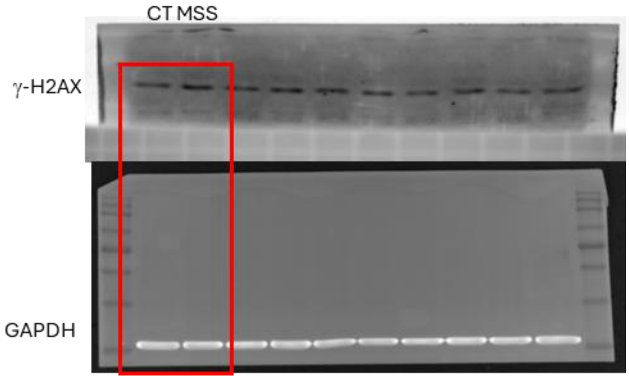

Figure 7A:

Western blot analysis of SOD1 in woozy mouse and wild type controls (wt).

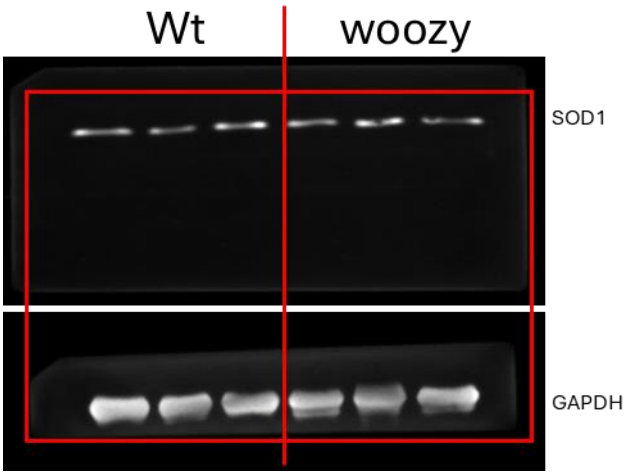

Figure 7B:

Western blot analysis of SOD2 in woozy mouse and wild type controls (wt).

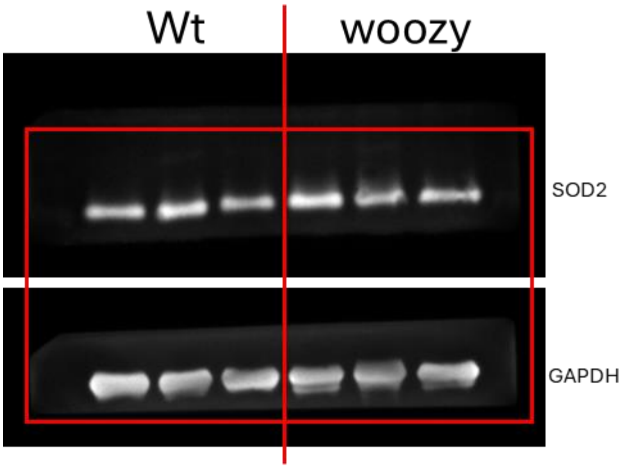

Figure 7C:

Western blot analysis of CAT in woozy mouse and wild type controls (wt).

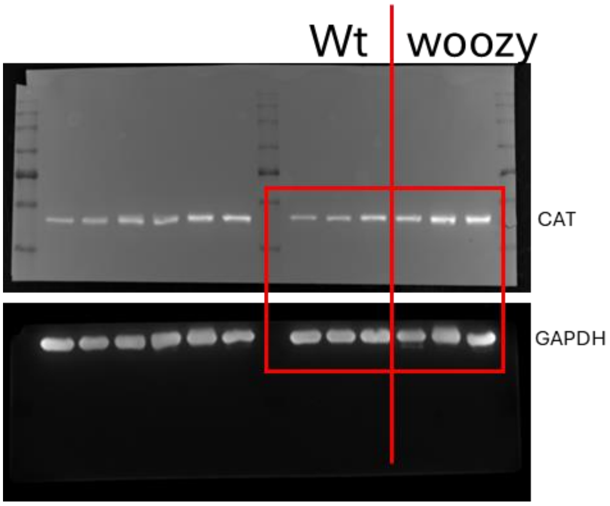

Supplementary figure 2:

Western blot analysis of mitofusin 2in patient-derived fibroblasts (MSS) and controls (CT).

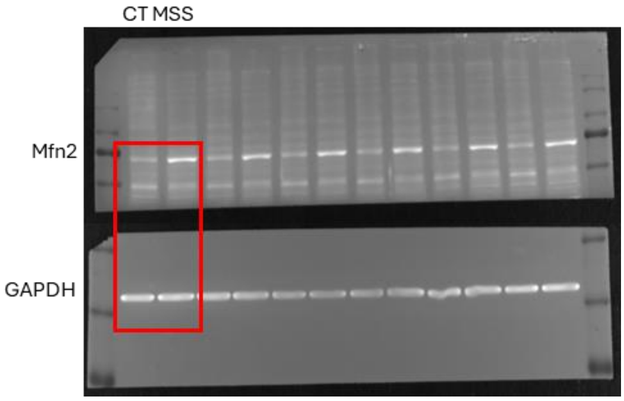

Supplement: Supplementary file 2 — Supplementary Material 2 [file 41598_2025_96467_MOESM2_ESM.pdf]
